# Proper use of light environments for mitigating the effects of COVID-19 and other prospective public health emergency lockdowns on sleep quality and fatigue in adolescents

**DOI:** 10.1016/j.heliyon.2023.e14627

**Published:** 2023-03-20

**Authors:** Peijun Wen, Fuyun Tan, Meng Wu, Qijun Cai, Ruiping Xu, Xiaowen Zhang, Yongzhi Wang, Shukun Li, Menglai Lei, Huanqing Chen, Muhammad Saddique Akbar Khan, Qihong Zou, Xiaodong Hu

**Affiliations:** a School of Physical Education, South China University of Technology, Guangzhou, 510641, China; b State Key Laboratory for Artificial Microstructure and Mesoscopic Physics, School of Physics, Peking University, Beijing, 100871, China; c Guangzhou Institute of Sport Science, Guangzhou, 510620, China; d Dongguan Institute of Optoelectronics, Peking University, Dongguan, 523808, China; e Center for MRI Research, Academy for Advanced Interdisciplinary Studies, Peking University, Beijing, 100871, China

**Keywords:** COVID-19, Emergency lockdowns, Residential light environment, Adolescent, Sleep quality, Fatigue

## Abstract

Coronavirus disease 2019 (COVID-19) remains a public health emergency of international concern, and some countries still implement strict regional lockdowns. Further, the upcoming 2023 Asian Games and World University Games will implement a closed-loop management system. Quarantine can harm mental and physical health, to which adolescents are more vulnerable compared with adults. Previous studies indicated that light can affect our psychology and physiology, and adolescents were exposed to the artificial light environment in the evening during the lockdown. Thus, this study aimed to establish and assess appropriate residential light environments to mitigate the effects of lockdowns on sleep quality and fatigue in adolescents. The participants were 66 adolescents (12.15 ± 2.45 years of age) in a closed-loop management environment, who participated in a 28-day (7-day baseline, 21-day light intervention) randomized controlled trial of a light-emitting diode (LED) light intervention. The adolescents were exposed to different correlated color temperature (CCT) LED light environments (2000 K or 8000 K) for 1 h each evening. The results for self-reported daily sleep quality indicated that the low CCT LED light environment significantly improved sleep quality (p < 0.05), and the blood test results for serum urea and hemoglobin indicated that this environment also significantly reduced fatigue (p < 0.05) and moderately increased performance, compared to the high CCT LED light environment. These findings can serve as a springboard for further research that aims to develop interventions to reduce the effects of public health emergency lockdowns on mental and physical health in adolescents, and provide a reference for participants in the upcoming Asian Games and World University Games.

## 1 Introduction

Coronavirus disease 2019 (COVID-19) remains a public health emergency of international concern, with more than 752 million confirmed cases and over 6.8 million deaths globally (last update: January 28, 2023; World Health Organization data). Some countries have implemented strict precautionary measures to prevent viral transmission and limit contact with COVID-19 patients. Some cities or communities remain locked down, and millions of people live in regions with closed-loop management systems or are required to stay at home. Although these preventative measures are critical to minimize the spread of COVID-19, lockdowns may affect people's mental and physical health, such as by increasing the risk of insomnia, depression, anxiety, fatigue, and eating disorders [[1], [2], [3]].

During COVID-19 lockdowns, people's lifestyles and interactions with others are completely altered [4]. Moreover, fear of infection aggravates anxiety [5]. These negative responses could be inextricably linked to quarantine measures [6]. A meta-analysis of nearly half a million people found that 40.5% of the global population has experienced sleep problems during the COVID-19 pandemic [7]. Another study indicated that Chinese participants experienced a 29.2% reduction in sleep quality while in quarantine [8]. Moreover, compared with adults, adolescents are more vulnerable to the negative effects of COVID-19 lockdowns [9]. There were 62% of children with sleep disorders and 55.6% of adolescents had sleep problems during the lockdowns [10,]. Sleep quality was deemed to be worse [11]. The COVID-19 pandemic and subsequent quarantine measures have had a detrimental impact on the mental and physical health of approximately 80% of adolescents [12]. In addition to the current pandemic, other prospective public health emergencies, such as the Monkeypox outbreak, could also cause large-scale lockdowns. Therefore, the effects of lockdowns on health need to be considered.

Exercise is an important activity in daily life and during the lockdown [13,14]; however, previous studies have indicated that COVID-19 lockdowns significantly affect fatigue and exercise performance [[15], [16], [17]], and young athletes have also been affected [18]. The 2021 Tokyo Summer Olympic Games and 2022 Beijing Winter Olympic Games were held using closed-loop management, in which all the participants trained, slept, and lived in isolation to prevent viral transmission. A closed-loop management policy will also be implemented at the upcoming 2023 Hangzhou Asian Games and 2023 Chengdu World University Games (Universidade). The impact of closed-loop management will affect tens of thousands of participants in these games, including athletes, coaches, referees, staff, journalists, and volunteers. Therefore, the purpose of this study was to simulate, establish and assess an appropriate, convenient, and replicable intervention to mitigate the effects of public health emergency lockdowns on sleep quality and fatigue in adolescents. Further, the results could be used as a reference for participants in the upcoming games.

Following the discovery of intrinsically photosensitive retinal ganglion cells in the early 21st century, an increasing number of researchers have realized that light has not only visual but also non-visual effects on humans [19]. The light intervention has been demonstrated to be among the most effective and safe physical methods for improving mental and physical health [20,21]. Adolescents are particularly susceptible to the effects of evening light environments [22]. Furthermore, with the development of solid-state lighting sources, light-emitting diode (LED) technology has been widely applied for display and illumination [23]. Nowadays, LEDs are among the most popular residential light sources. Changing LED parameters, such as correlated color temperature (CCT) and wavelength, can create different light environments to achieve the objective of this study which aimed to establish and evaluate the different types of evening light environments to mitigate the effects of COVID-19 lockdown on sleep quality and fatigue in adolescents. Our findings could be expediently applied in adolescents' daily lives to improve their sleep quality and physical health, and has implications for the participants under closed-loop management. In this study, we conducted a randomized controlled trial of different CCT LED light environments with adolescents under 28-day closed-loop management, including a 7-day baseline and 21-day light intervention. In particular, we focused on the effects of light on adolescents’ sleep quality and fatigue.

## 2 Material and methods

### 2.1 Research design

This study conducted a randomized controlled trial with pre-test, mid-test and post-test design. This type of research required two independent groups which were set as two different major types of light environments in this study. They were the low CCT light environment and high CCT light environment. The light intervention lasted for 21 days, and the measurements included subjective and objective tests. Besides, in order to mitigate the effects of the COVID-19 lockdown on sleep quality and fatigue in adolescents, and provide a reference for the participants in the upcoming 2023 Asian Games and World University Games, this study recruited the participants from the Guangzhou swim team once they qualified for the requirements.

### 2.2 Participants

Participants in this study included 66 healthy adolescents. Inclusion criteria for the trial were as follows: (1) taking no medication for sleep, circadian rhythm, fatigue, or mental health for three months prior to the study; (2) having no sleep or light intervention experiment experience; and (3) having long-term exercise habits, defined as exercising for at least five days a week for three months prior to the study. The 66 adolescents were randomly assigned (1:1) to either the low or high CCT group. Table 1 shows participant characteristics. No significant differences were observed in gender, age, or body mass index between the two groups (p > 0.05).

**Table 1:** Participants’ demographic characteristics (Mean ± SD).

	Low CCT group	High CCT group
Number of people	n = 33	n = 33
Gender	14 female, 19 male	14 female, 19 male
Age (years)	12.15 ± 2.39	12.15 ± 2.51
Tanner stages-no.		
2	10	10
3	17	17
4	6	6
Body mass index	18.9 ± 2.39	18.7 ± 2.50

**Fig. 1:**
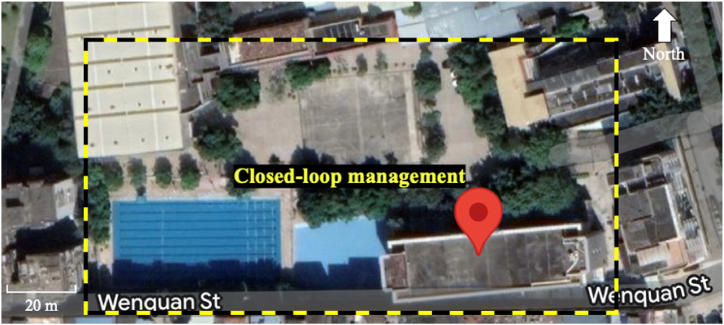
A map of the location of the experiment. All adolescent participants remained in a closed-loop management environment and followed the same daily schedule during this 28-day trial. They lived in the building indicated with a red pin on the map, which had classrooms, playrooms, bedrooms, a canteen, a fitness room, and an indoor swimming pool.

All participants remained in a closed-loop management environment for the duration of the study (Fig. 1). They all followed the same daily schedule during the 28-day trial (7-day baseline and 21-day light intervention). The daily schedule in this study restored their activities of daily living. They awoke at 07:00 and attended classes in the morning, participated in the same exercises (e.g., swimming or strength training under coach instruction) in the afternoon, completed their homework in the evening, and received a 1-h light intervention before bedtime (22:00). All participants also had identical diets every day during the trial, and did not drink coffee or tea. They stayed in the same quiet bedrooms, which had curtains to reduce their exposure to sunlight prior to waking, and air conditioners to maintain a room temperature of 26 °C. Aside from the light intervention, all participants were exposed to the same residential fluorescent light sources. Thus, given the participants’ consistent living conditions, variables affecting sleep quality and fatigue were controlled in this study.

### 2.3 Light environments and light intervention design

**Fig. 2:**
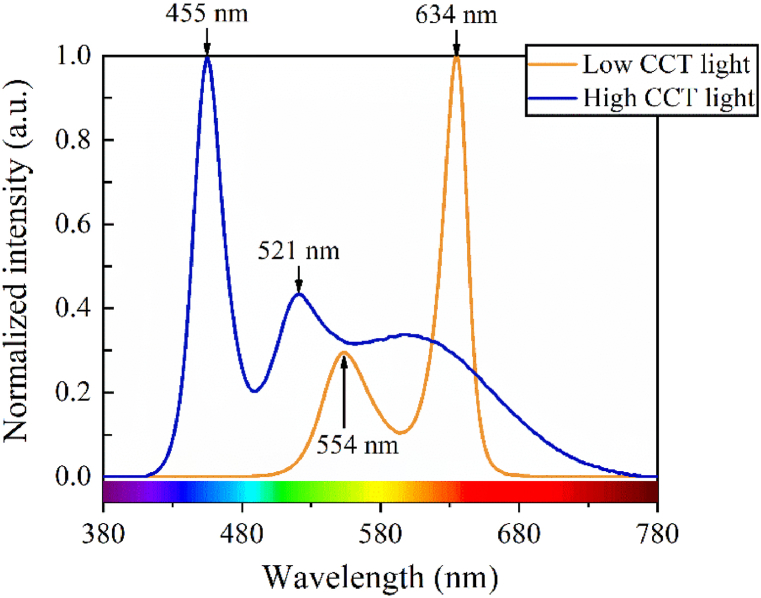
The spectra of the low CCT light and high CCT light.

LEDs are among the most commonly used forms of residential lighting in this century, its advantages are the long lifespan, environmentally safe, higher energy efficiency and design flexibility when compared to traditional lighting solutions (such as the incandescent lamp and fluorescent lamp). Thus, we designed two CCT LEDs as the light sources for the intervention: the low CCT light at 2000 K and the high CCT light at 8000 K (Fig. 2). The primary design philosophy of the low CCT light was that it avoided short-wavelength light, such as blue light, and minimized circadian rhythm stimulation. The low CCT light was calculated by measuring the circadian light (CL_A_) and circadian stimulus (CS) [24,25]. Therefore, the low CCT light was mixed with innovative green and red LEDs, which not only had low CCT but also reached a high color reading index (80). For the high CCT light, the blue, green, and red LEDs were mixed to form a classic white LEDs spectrum. These two light environments were considered safe and met the IEC 62471:2006 standard (Table 2).

**Table 2:** Parameters of the light environments.

	Low CCT light	High CCT light
*Alpha-opic equivalent daylight (D65) illuminance (lx)*		
*S*-cone-opic	2.26	254.06
M-cone-opic	117.69	209.00
l-cone-opic	183.54	208.81
Rhodopic	50.49	216.53
Melanopic	27.81	220.60
Illuminance	200	200
*Light intervention information*		
CCT	2000 K	8000 K
E_B_	0.003 W/m^2^	0.221 W/m^2^
CL_A_	54.74	361.92
CS	0.079	0.353
Duration	1 h/day	1 h/day
Period	21 days	21 days

**Fig. 3:**
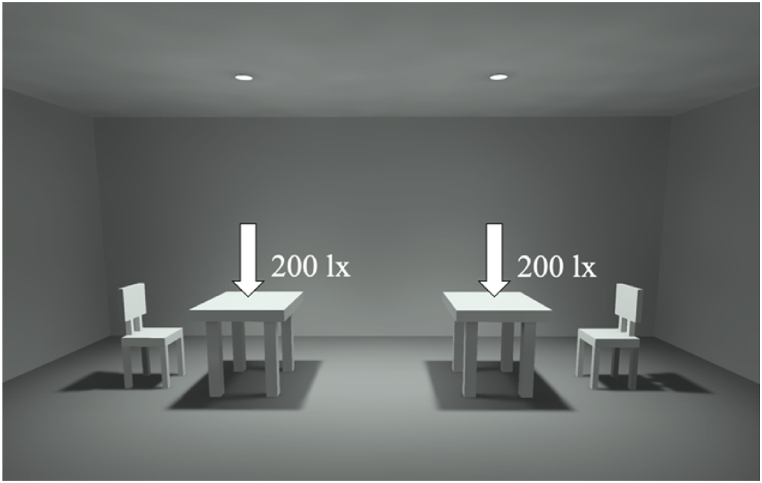
A schematic diagram of the light intervention rooms. The desks were 80 cm in height. The arrows indicate where the illuminance was measured. Both the high CCT light room and low CCT light room were maintained at the same illuminance (200 lx).

Two playrooms in the building (red pin on the map, Fig. 1) were refitted to serve as the experimental rooms. Low and high CCT lights were separately installed in the two rooms (Fig. 3). The illuminance of the two experimental rooms was the same, we set that at 200 lx because this illuminance was close to the actual residential light environment in the evening and also sufficient for reading. The participants underwent a 21-day light intervention and were exposed to different CCT light environments for 1 h (20:45–21:45) every night before sleep.

During the light intervention, participants did their homework, but were not permitted to carry or use electronic devices. This guaranteed that no other illuminants were present in the experimental rooms. Table 2 presents information on the light environments and intervention. In this study, the participants in the different CCT groups were only exposed to their own light condition during the 1-h light intervention in the evening in the whole experiment. They went to sleep after the light intervention. The only difference between the two groups was the light environment before sleep, and the illuminance and other variables were controlled as presented in section 2.2. Therefore, the differences in the measurements were attributed to the different light environments.

### 2.4 Measurements

**Fig. 4:**
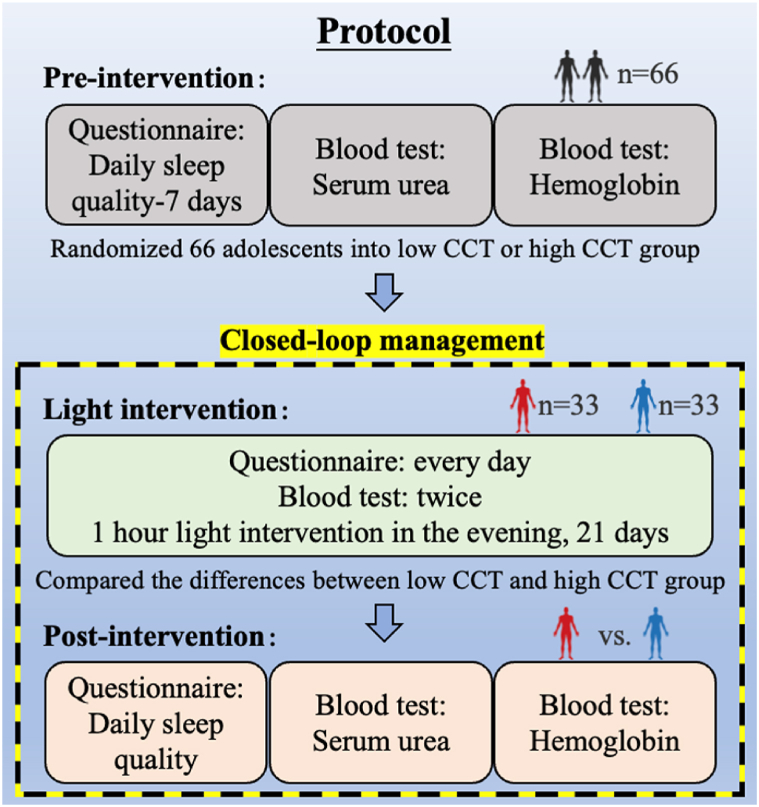
The experimental process of the 28-day study protocol.

We investigated how different evening light environments affect sleep quality, fatigue, and hemoglobin in a closed-loop management environment. The experimental results and statistical analyses were based on self-report questionnaires and blood tests (Fig. 4).

**Sleep quality**. Participants recorded their daily sleep quality in the morning after waking using a simple self-report questionnaire on sleep quality. The questionnaire provided five options participants could select to report their perceived sleep quality from the previous night: very bad, bad, neither bad nor good, good, and very good. These five options correspond to scores ranging from one to five. Participants began recording their sleep quality seven days prior to the light intervention (baseline), and then continued to record their sleep quality daily during the light intervention (21 days). A simple daily sleep quality record is not only a convenient and accessible measurement for young adolescents (as young as 10 in the present study) but also beneficial for tracking their daily sleep quality tendencies.

**Serum urea**. Urea in the blood is the final product of protein metabolism and among the most critical biological indicators that reflect one's degree of fatigue [26]. In this study, all participants took part in exercise training 2 h a day for three months before and during the light intervention. We hypothesized that different light environments would directly affect sleep quality and indirectly affect fatigue recovery. Participants' serum urea concentrations were measured using a urea kit (UV-GLDH method, Shanghai Kehua Bio-engineering Corporation, China) that could be used for the quantitative determination of urea in participants' serum. The serum urea concentration is proportional to fatigue.

**Hemoglobin.** Hemoglobin is a protein that carries oxygen from the lungs to the tissues of the body, improves blood flow to working muscles, and plays a critical role in endurance sports. Hemoglobin concentrations can indicate aerobic capability and prevent sports anemia [27]. Therefore, the test of hemoglobin concentration is a classical indicator that shows one's potential performance in aerobic exercise. Participants' hemoglobin concentrations were measured using the SULFOLYSER SLS-211 A (SLS-Hb method, Sysmex Corporation, Japan). The hemoglobin concentration is positively correlated with sports performance.

Over the course of the study, participants were given four blood tests, each scheduled at 08:00, to measure their serum urea and hemoglobin concentrations. The first blood test before the light intervention was recorded as the baseline measurement. The second and third blood tests were performed during the light intervention, and the fourth blood test was performed the day after the final night of the light intervention.

### 2.5 Statistical analysis

Statistical analyses were conducted using SPSS (IBM Corporation, USA). First, we performed a Gaussian distribution test (Shapiro-Wilk) to analyze all the data from the self-report questionnaires and blood tests. Next, a two-way (group: low CCT and high CCT; time: four blood tests) repeated measures ANOVA was used to analyze the results. Post hoc tests were corrected for multiple comparisons. We particularly focused on the group × time interaction effect and group effect. Statistical significance was set at p < 0.05, and an effect size (partial eta squared: $\eta_{p}^{2}$) greater than 0.14 were considered a large effect.

### 2.6 Ethical considerations

The Ethics Committee of the Guangzhou Institute of Sport Science evaluated and authorized this study and its trial protocols, which adhered to the principles of the Declaration of Helsinki. The nurses from Longdong Hospital of Tianhe District, Guangzhou helped to draw the participants' blood. All the blood-test procedures qualified for the guideline of the National Health Commission of the People's Republic of China.

## 3 Results

**Fig. 5:**
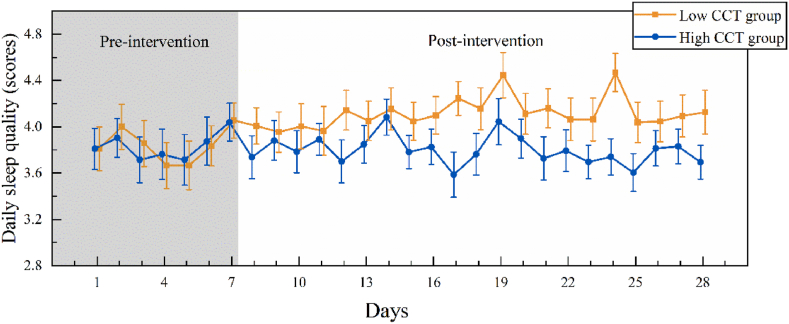
The daily sleep quality of the two groups before and during the light intervention (Mean ± SEM). No significant between-group differences were observed for daily sleep quality prior to the light intervention (p = 0.953). However, the low CCT group reported significantly better sleep quality compared with the high CCT group in the post-intervention (p = 0.029, $\eta_{p}^{2}$ = 0.218).

Higher self-reported sleep quality scores were considered to indicate better sleep quality (Fig. 5 and Table S1). Neither a significant group × time interaction effect (p = 0.967) nor a group effect (p = 0.953) was observed at baseline. The average sleep quality scores were 3.84 (low CCT group) and 3.83 (high CCT group). However, we observed both a group × time interaction effect (p = 0.045, $\eta_{p}^{2}$ = 0.075) and a group effect (p = 0.029, $\eta_{p}^{2}$ = 0.218) during the light intervention period (21 days). The sleep quality of the low CCT group was superior to that of the high CCT group on the first day of the light intervention, and was invariably better for the low CCT group compared with the high CCT group during the entire 21-day light intervention period. The average sleep quality score of the low CCT group was 4.12, while that of the high CCT group remained at 3.80. Therefore, participants in the low CCT group reported better subjective sleep quality than did those in the high CCT group after the light intervention.

**Fig. 6:**
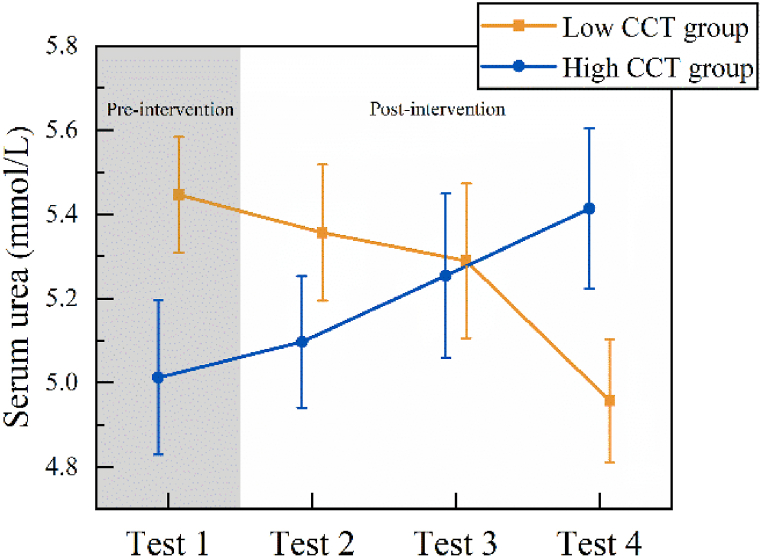
The serum urea results of the low CCT group and high CCT group (Mean ± SEM). A group × time interaction effect on serum urea concentration was observed (p = 0.011, $\eta_{p}^{2}$ = 0.162). Post-intervention, serum urea concentration decreased in the low CCT group, but increased in the high CCT group. Therefore, the low CCT light was observed to reduce fatigue after performing the same exercise routine.

Regarding the blood test results, we observed a group × time interaction effect (p = 0.011, $\eta_{p}^{2}$ = 0.162) for serum urea concentration (Fig. 6 and Table S2). We compared the two groups using post-hoc statistics. Prior to the light intervention, serum urea concentration in the low CCT group was 0.434 mmol/L higher than that in the high CCT group; however, this difference was not significant (p = 0.062). Results for the second (p = 0.314) and third (p = 0.895) blood tests also did not significantly differ between groups. In the final blood test, serum urea concentration in the low CCT group was 0.456 mmol/L lower than that in the high CCT group, and this difference was statistically significant (p = 0.049, $\eta_{p}^{2}$ = 0.172). Participants performed the same exercises daily for three months before and during the light intervention. A lower serum urea concentration indicated better fatigue recovery. Hence, the low CCT light was found to reduce fatigue in adolescents.

**Fig. 7:**
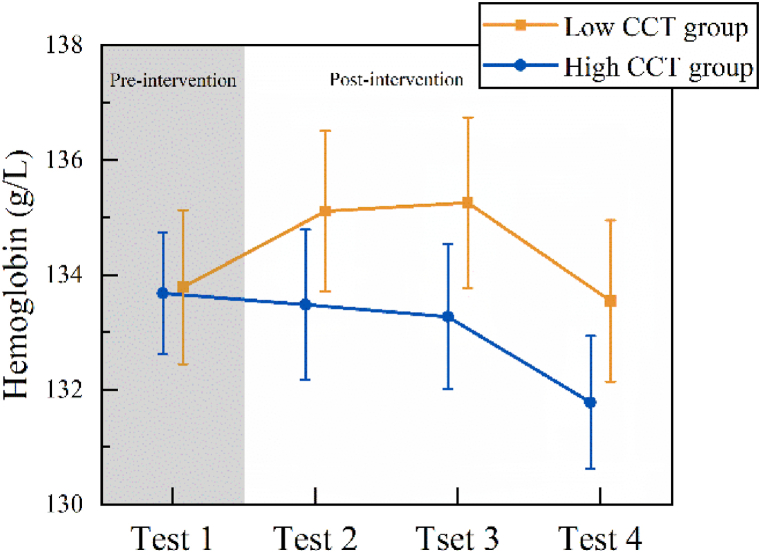
The hemoglobin results of the low CCT group and high CCT group (Mean ± SEM). The low CCT group had a better tendency than the high CCT group, but this difference was not significant (p = 0.449).

The hemoglobin results did not indicate a group × time interaction effect or a group effect (Fig. 7 and Table S3). Before the light intervention, hemoglobin concentration was similar between both groups (low CCT group: 133.8 g/L, high CCT group: 133.7 g/L). However, hemoglobin concentration in the low CCT group increased to 135.1 g/L and 135.3 g/L in the second and third blood tests, respectively, and decreased to 133.5 g/L in the final blood test. Hemoglobin concentration in the high CCT group declined after the light intervention and consistently remained lower than that of the low CCT group. A higher hemoglobin concentration demonstrates better aerobic capacity; however, this difference was not statistically significant (p = 0.449).

## 4 Discussion

The COVID-19 pandemic has greatly affected people's daily lives. Although quarantine can reduce viral transmission, it also affects both mental and physical health. Our study assessed the effects of different light environments on sleep and fatigue in adolescents within a long-term closed-loop management environment. The 28-day trial simulated the most severe quarantine period. Moreover, the upcoming 2023 Asian Games and World University Games will implement closed-loop management systems. Accordingly, the aim of this study was to establish and assess an appropriate residential light environment to reduce the impact of COVID-19 and other prospective public health emergency lockdowns on sleep quality and fatigue in adolescents. The results could also provide a reference for participants in the upcoming Asian Games and World University Games.

The results on daily sleep quality revealed that adolescents exposed to the low CCT light environment in the evenings reported better sleep quality than those in the high CCT light environment. Although the adolescents remained in a closed-loop management environment in this study, these sleep quality results were similar to those of previous studies without closed-loop management. Studies on children [28] and adolescents [29] have shown that exposure to a high CCT light environment in the evening could affect sleep quality and reduce sleepiness, compared with a low CCT light environment. Previous studies have further indicated that high CCT light could affect circadian rhythm [[30], [31], [32]] and influence sleep quality [33]. Further, in the current study, of the positive effect of low CCT light on sleep quality in adolescents was significant and prominent from the first day of the light intervention. We observed a fast response to the light environment, and adolescents were sensitive to it in the evening. These findings on adolescents are similar to those of previous studies on adults, the light environments within the 3 h before sleep was highly associated with sleep quality [34]. For example, previous studies reported that only 15 s of high-intensity light might shift the circadian pacemaker [35], while 30 min of dim light (30 lx) exposure at home also suppressed 15% melatonin among women [36], and 1 h of light exposure was shown to be enough to affect the human circadian clock [37,38].

The main difference in the spectra between the two light environments in this trial is the proportion of blue light; the low CCT light did not include blue light; however, the high CCT light did. The spectrum of the high CCT light in this study was a conventional residential white-light LED spectrum. Generally, residential white-light LEDs are created using one of two methods. The first method uses LEDs that include blue light to emit fluorescent powder. The second method combines disparate monochromatic LED lights, such as red, green, and blue light. Therefore, a high CCT light environment must have a blue-light component. Previous studies have indicated that short-wavelength-enriched light can severely suppress melatonin and delay the circadian rhythm phase in the evening [33,[39], [40], [41]]. Adolescents in the low CCT group likely had better sleep quality because the low CCT light included no blue light at all. Although this study's participants were adolescents, the effects of light on the present sample seemed to be the same as those found in adults. In addition, the type of light source appears to be unimportant, because a previous study showed that, compared with a traditional fluorescent lamp (6000 K), low CCT LEDs (2000 K) had a better influence on male adolescents' sleep quality (12.8 ± 1.7) [29]. In this study, the low CCT LEDs (2000 K) also revealed a more positive effect on the sleep quality of both male and female adolescents compared with high CCT LEDs (8000 K). Therefore, we conclude that the crucial factor affecting adolescents' sleep quality is a CCT-light environment, rather than the type of luminaires.

In addition to sleep quality, we evaluated the effects of lockdowns on fatigue and performance in adolescents. During exercise, protein is decomposed into amino acids as the energy supply through deamination when glycogen and fat cannot meet the energy demand of exercise, increasing urea levels in the blood. In this study, adolescents exercised daily during the trial, and serum urea levels were evaluated. The results showed that adolescents who were exposed to the low CCT light environment had better fatigue recovery than did those exposed to the high CCT light environment. Previous studies on melatonin suppression could explain this finding. The high CCT light had a blue light component, and its peak wavelength was 455 nm, which can significantly affect circadian rhythm and sleep quality by suppressing melatonin [39,42]. Thus, the low CCT group likely had better fatigue recovery than the high CCT group because the low CCT group had good sleep quality.

Further, oxygen consumption and use increase significantly during aerobic exercise, which produces free radicals that can lead to muscle fatigue and damage. Melatonin acts as an antioxidant that neutralizes harmful oxidative radicals [43,44]. In addition, melatonin plays a role in regulating the immune system, which helps improve the body's ability to resist or eliminate potential foreign substances [45]. The previous study showed that female patients who received oral melatonin significantly decreased their levels of fatigue [46]. Therefore, melatonin is not only related to sleep quality but can also affect fatigue at the same time. In this study, the low CCT light had no blue light, but instead only combined green and red LEDs. Therefore, low CCT light can prevent circadian rhythm stimulation, and a low CCT light environment may increase melatonin secretion [47]. In addition, an effective method to reduce fatigue is photobiomodulation (red or near-infrared light, from 660 nm to 950 nm), which is a low-level laser or LED therapy [[48], [49], [50]]. In this study, the peak wavelength of the low CCT light was 634 nm, and serum urea results indicated that the low CCT group had significantly decreased fatigue after the light intervention. This finding was consistent with Batoni et al. who reported that 660 nm and 850 nm LED therapy can increase performance [51]. Another study also found that 630 nm LED therapy improved muscle recovery after exercise [52]. Therefore, in this study, although the intensity of the low CCT light environment was not as strong as that of traditional photobiomodulation, it still helped adolescents experience reduced fatigue after exercise.

Hemoglobin is also an important index that reflects physical condition and performance. Hemoglobin concentration significantly affects cardiopulmonary exercise performance, which is related to oxygen. A previous study conducted with adults showed that high CCT light could increase peak O_2_ uptake and reduce muscle fatigue [53]. In addition, hemoglobin concentration is associated with endurance performance, Zhao et al. found that high CCT light treatment improved the long-distance running performance of basketball players [54], and a similar result of improvement of physical endurance was found in an experiment on mice as well [55]. Although hemoglobin concentrations did not significantly differ between the two groups in this study, we observed that the hemoglobin concentration in the low CCT group was always higher than that in the high CCT group after the light intervention. These findings may be helpful not only for adolescents but also for professional athletes, because the upcoming 2023 Asian Games and World University Games will implement closed-loop management, and some previous studies reported that Olympians were previously negatively affected by closed-loop management [56,57].

Although this study employed a randomized controlled trial to test the effects of different CCT light environments on sleep quality and fatigue in adolescents under closed-loop management, it was not without limitations. Sleep quality was measured using a self-report questionnaire, while the ideal situation would be to monitor sleep quality using actigraphy or polysomnography in a laboratory setting. Further, although we discussed that melatonin and circadian rhythm could be responsible for the differences observed post-intervention, this is only based on the results of previous studies, and it would be preferable if we could examine the effects of melatonin and circadian rhythm in real time. Besides, fewer studies are available on adolescents compared with those on adults, and we hope more researchers will focus on adolescent health in future studies.

Finally, our findings provide evidence that proper use of light environments could be a positive treatment to mitigate the effects of COVID-19 lockdown on sleep quality and fatigue in adolescents. Therefore, the organizers and participants of the 2023 Asian Games and World University Games could consider using the dynamic and intelligent lighting system to reduce the effects of closed-loop management.

## 5 Conclusions

In this study, we evaluated appropriate residential light environments to mitigate the effects of COVID-19 lockdowns on sleep quality and fatigue in adolescents. Compared with a high CCT light environment (8000 K), exposure to a low CCT light environment (2000 K) in the evening was shown to significantly improved sleep quality and reduced fatigue during 21-day closed-loop management. Our findings could be applied to quarantine and should have important implications for understanding the impact of light on adolescent health. The COVID-19 pandemic remains a public health emergency of international concern. Therefore, increasing the number of studies on specialized interventions for adolescents and other vulnerable populations in the context of a health crisis is critical.

## Author contribution statement

Peijun Wen: conceived and designed the experiments; performed the experiments; analyzed and interpreted the data; contributed reagents, materials, analysis tools or data; wrote the paper.

Fuyun Tan: performed the experiments; analyzed and interpreted the data. Meng Wu: contributed reagents, materials, analysis tools or data.

Qijun Cai: performed the experiments. Ruiping Xu: performed the experiments. Xiaowen Zhang: performed the experiments. Yongzhi Wang: contributed reagents, materials, analysis tools or data. Shukun Li: analyzed and interpreted the data.

Menglai Lei: analyzed and interpreted the data.

Huanqing Chen: contributed reagents, materials, analysis tools or data.

Muhammad Saddique Akbar Khan: contributed reagents, materials, analysis tools or data.

Qihong Zou: contributed reagents, materials, analysis tools or data. Xiaodong Hu: conceived and designed the experiments; contributed reagents, materials, analysis tools or data.

## Funding statement

This work was supported by 10.13039/501100001809National Natural Science Foundation of China [12074129, 81871427]; Fundamental Research Funds for the Central Universities [2022ZYGXZR104]; Beijing United Imaging Research Institute of the Intelligent Imaging Foundation [CRIBJZD202101].

## Data availability statement

Data will be made available on request.

## Declaration of interest's statement

The authors declare no conflict of interest.

## Additional information

Supplementary content related to this article has been published online at [URL].
