# Supplementary material for: Proper use of light environments for mitigating the effects of COVID-19 and other prospective public health emergency lockdowns on sleep quality and fatigue in adolescents
Source: Heliyon. 2023 Mar 20;9(4):e14627. doi: 10.1016/j.heliyon.2023.e14627 (PMC10027303; doi:10.1016/j.heliyon.2023.e14627)
Supplement: Multimedia component 1 [file mmc1.docx]

**Supplementary material**

We particularly focused on the group × time interaction effect and group effect in this study.

**Table S1.** Self-reported daily sleep quality results (F/p)

|  | Group×Time $(\eta_{p}^{2}$) | Group $(\eta_{p}^{2}$) | Time  $(\eta_{p}^{2}$) |
| --- | --- | --- | --- |
| Pre-intervention (7 days) | 0.227/0.967 (0.011) | 0.004/0.953 (<0.001) | 2.402/0.032 (0.107) |
| Post-intervention (21 days) | 1.617/**0.045** (0.075) | 5.560/**0.029** (0.218) | 1.881/0.013 (0.086) |

**Table S2.** Summary of serum urea post hoc statistics

|  |  | F | p | $\eta_{p}^{2}$ |
| --- | --- | --- | --- | --- |
| ***Group×Time*** |  | 4.060 | **0.011** | 0.162 |
| ***Group*** | | | |  |
| Low test 1 vs. High test 1 | | 3.895 | 0.062 | 0.156 |
| Low test 2 vs. High test 2 | | 1.064 | 0.314 | 0.048 |
| Low test 3 vs. High test 3 | | 0.018 | 0.895 | 0.001 |
| Low test 4 vs. High test 4 | | 4.377 | **0.049** | 0.172 |
| ***Time*** | | | |  |
| Low CCT light | | 2.956 | 0.039 | 0.123 |
| High CCT light | | 1.302 | 0.282 | 0.058 |

Low test 1: low CCT group test 1. High test 1: high CCT group test 1.

**Table S3.** Hemoglobin results

|  | F | p | $\eta_{p}^{2}$ |
| --- | --- | --- | --- |
| Group×Time | 0.951 | 0.420 | 0.034 |
| Group | 0.591 | 0.449 | 0.021 |
| Time | 2.747 | 0.048 | 0.092 |
|  |  |  |  |
